# Supplementary material for: Predicting visual function by interpreting a neuronal wiring diagram
Source: Nature. 2024 Oct 2;634(8032):113–23. doi: 10.1038/s41586-024-07953-5 (PMC11446822; doi:10.1038/s41586-024-07953-5)
Supplement: Supplementary file 6 — CRF and ERF predictions for individual TmY4 and TmY9 cells. Analogous to Supplementary Data 3, but for TmY target types. Shown are the top four monosynaptic pathways, the strongest pathway passing through each of the top ten intermediary types (ranking from Extended Data Fig. 7), and the trisynaptic pathway Tm1–TmY–Dm3–TmY (see the section entitled Prediction of spatial normalization). [file 41586_2024_7953_MOESM6_ESM.zip › DataS4/TmY9qΓèÑ/720575940616897876.pdf]

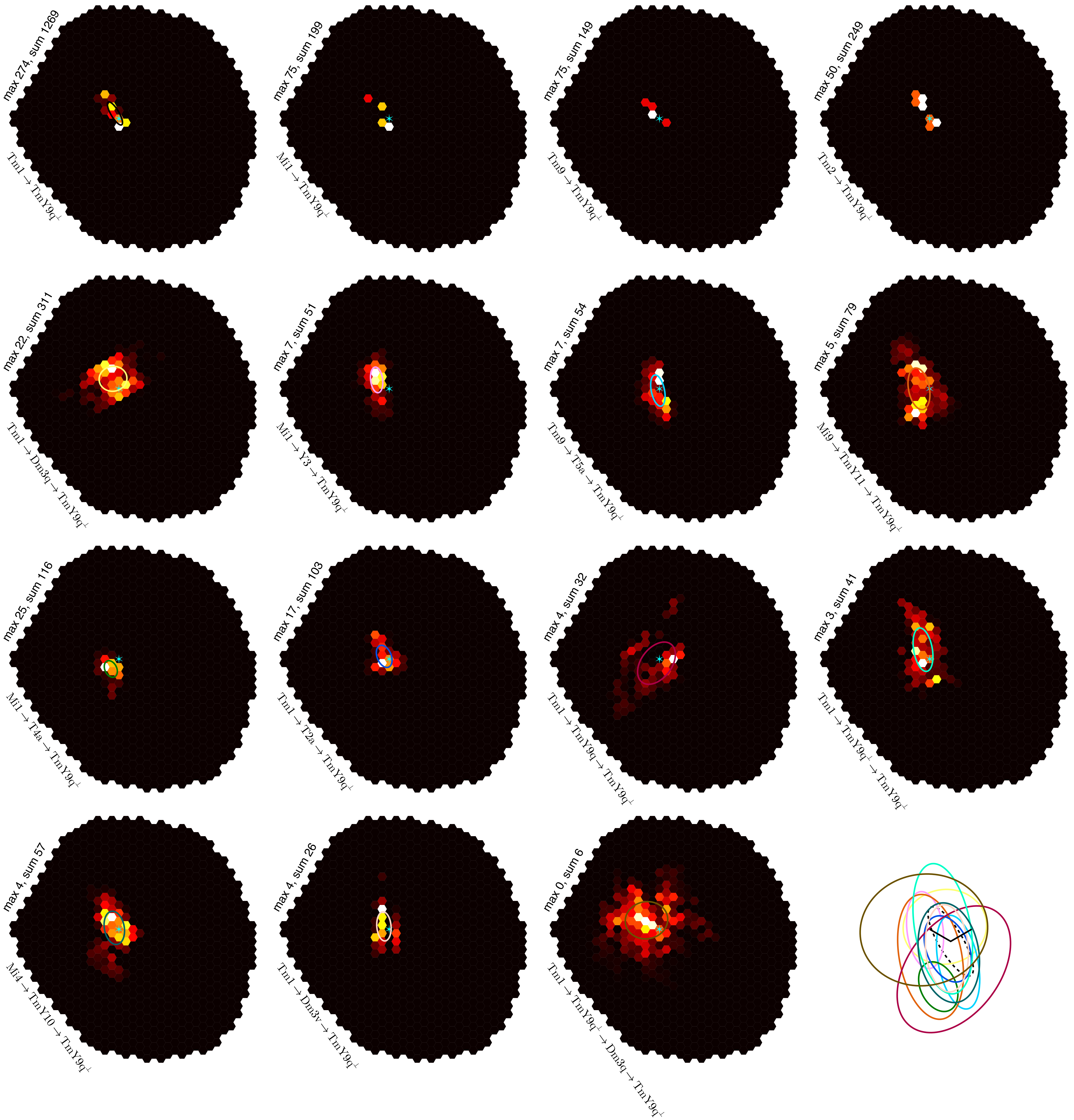
max 274, sum 1269
Tm1 → TmY9q⊥
max 75, sum 199
Mi1 → TmY9q⊥
max 75, sum 149
Tm9 → TmY9q⊥
max 50, sum 249
Tm2 → TmY9q⊥
max 22, sum 311
Tm1 → Dm3q → TmY9q⊥
max 7, sum 51
Mi1 → Y3 → TmY9q⊥
max 7, sum 54
Tm9 → T5a → TmY9q⊥
max 5, sum 79
Mi9 → TmY11 → TmY9q⊥
max 25, sum 116
Mi1 → T4a → TmY9q⊥
max 17, sum 103
Tm1 → T2a → TmY9q⊥
max 4, sum 32
Tm1 → TmY9q → TmY9q⊥
max 3, sum 41
Tm1 → TmY9q⊥ → TmY9q⊥
max 4, sum 57
Mi4 → TmY10 → TmY9q⊥
max 4, sum 26
Tm1 → Dm3v → TmY9q⊥
max 0, sum 6
Tm1 → TmY9q⊥ → Dm3q → TmY9q⊥
